# Desmoplastic Small Round Cell Tumors Presented With ST-Segment Elevation Myocardial Infarction and Cardiac Tamponade in a Young Adult Female: A Case Report

**DOI:** 10.7759/cureus.53333

**Published:** 2024-01-31

**Authors:** Kensuke Takaoka, Ashwin Venkataraman, Clarke Morihara, Arvin Tan, Witina Techasatian

**Affiliations:** 1 Internal Medicine, University of Hawaii at Manoa John A. Burns School of Medicine, Honolulu, USA; 2 Internal Medicine, Tripler Army Medical Center, Honolulu, USA

**Keywords:** pericardial effusion with cardiac tamponade, mass lesion lung, aggressive sarcoma, st-elevation myocardial infarction (stemi), desmoplastic small round cell tumors

## Abstract

Desmoplastic small round cell tumors (DSRCT) are very rare and aggressive diseases typically present with abdominal or retroperitoneal masses. We present a case of a young female who presented with ST-segment elevation myocardial infarction and cardiac tamponade and who was found to have DSRCT. The patient was coded at the emergency department. Left heart catheterization showed normal coronary arteries, and pericardiocentesis removed 1,260 mL of bloody pericardial effusions. The patient was stabilized, and a positron emission tomography scan revealed left intrahilar, hilar, and cardiophrenic masses with associated hypermetabolic right hilar, left hilar, subcarinal, costophrenic, aortopulmonary, paratracheal, and mediastinal lymphadenopathy. Cardiac magnetic resonance imaging showed multiple masses visualized in the pericardium, one mass anterior to the right ventricular outflow tract/pulmonary artery, and a second mass adjacent to the right ventricular apex. Computed tomography abdomen/pelvis showed no evidence of metastatic malignancy in the abdomen/pelvis. A biopsy of lung mass and lymph nodes showed desmoplastic small round cell tumors with sarcoma fusion gene detected (Ewing sarcoma RNA-binding protein 1-Wilms’ tumor 1). We performed cycle 1 of chemotherapy, including doxorubicin, vincristine, and cyclophosphamide, and the patient was transferred to an oncology center for further care. This case suggested that one of the differential diagnoses of lung and pericardium masses at a young age can be desmoplastic small round cell tumors. This case also highlighted that ST-segment elevation myocardial infarction can be secondary to neoplasm, especially at a young age besides myocardial infarction.

## Introduction

Desmoplastic small round cell tumors (DSRCT) are malignant neoplasms that are rare and aggressive [1], which were first reported in 1987 [2]. Typically arising from the serosa of the abdominal cavity, they are prevalent among adolescents and young adults with a male predominance. These tumors are characterized by clusters of small round cells surrounded by a desmoplastic stroma [3]. Its hallmark is the translocation of the Ewing sarcoma RNA-binding protein 1 gene (EWSR1) andWilms’ tumor 1 suppressor gene (WT1) that upregulates the expression of platelet-derived growth factor receptor α, vascular endothelial growth factor, and insulin-like growth factor 1 [3,4]. Treatment includes chemotherapy, radiation, and cytoreductive surgery with intra-peritoneal hyperthermic chemotherapy [1]. Chemotherapy usually consists of a combination of vincristine, doxorubicin, and cyclophosphamide alternating with ifosfamide and etoposide [5]. Despite prompt diagnosis and treatment, prognosis is poor due to the high rates of recurrence. The overall five-year survival was reported to be 33.3% [3]. We herein discuss a case of DSRCT presented with ST-segment elevation myocardial infarction (STEMI) and cardiac tamponade.

## Case presentation

**Figure 1:**
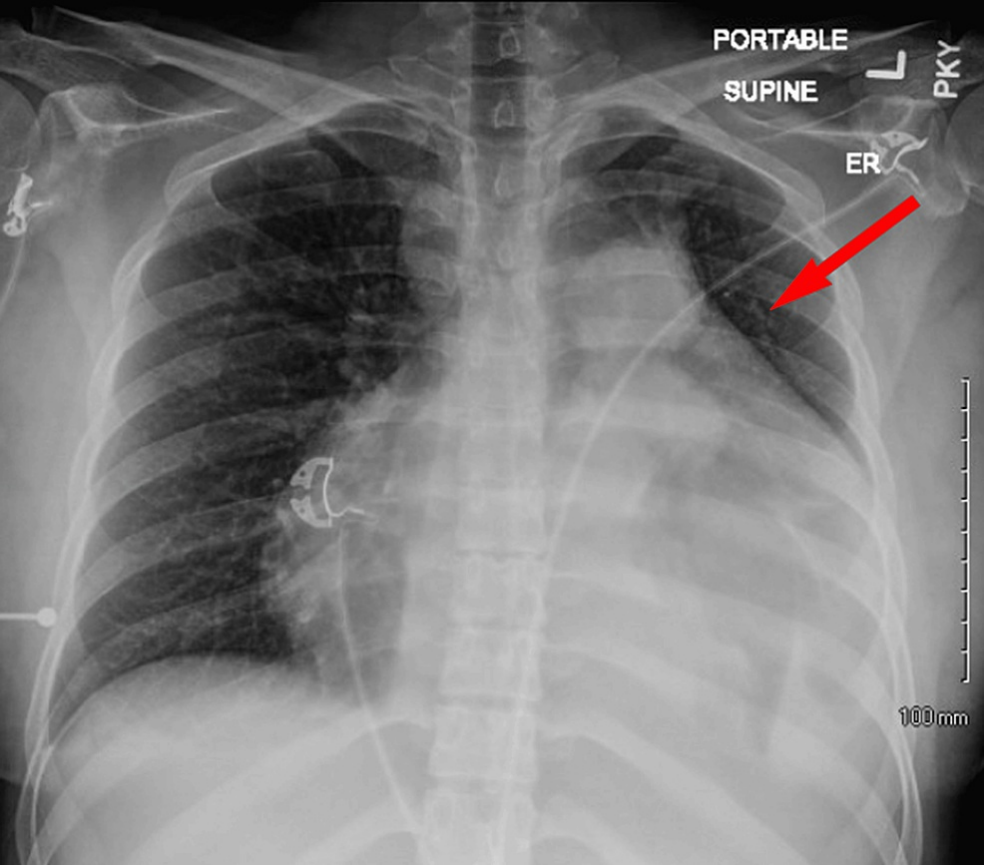
Chest x-ray on admission Cardiomegaly was noted with left retrocardiac opacity.

The patient is a 24-year-old female with no notable past medical history who presented to the emergency department (ED) with a complaint of progressive dyspnea on exertion, fatigue, abdominal pain, and back pain. The patient reported initial symptoms of fatigue that started six months prior to presentation, with dyspnea beginning two months prior to presentation, initially just with exertion, but progressing to dyspnea at rest on presentation. Of note, the patient is an airline First Officer and otherwise active and without any other medical co-morbidity. The patient denies any prior smoking history and endorsed being an avid surfer. The patient had presented to an urgent care prior to ED arrival at which time, and she was diagnosed with asthma and acute bronchitis and discharged with a six-day tapering course of oral corticosteroids, as well as oral azithromycin, which failed to improve symptoms. The patient presented to ED due to unremitting, progressive symptoms. Chest x-ray showed an enlarged cardiac silhouette (Figure 1).

**Figure 2:**
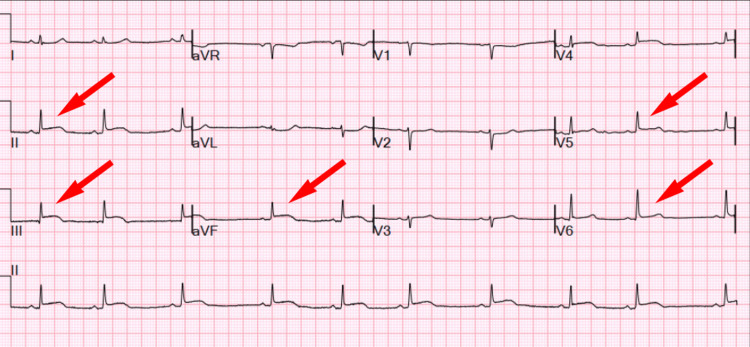
Electrocardiogram on admission Poor R wave regression and inferolateral ST-elevation were noted.

Due to symptoms of chest and abdominal pain, computed tomographic angiography dissection protocol was performed, which revealed no dissection, and a large pericardial effusion and left lung mass. An electrocardiogram (EKG) revealed ST-segment elevations in the inferolateral leads (Figure 2).

**Figure 3:**
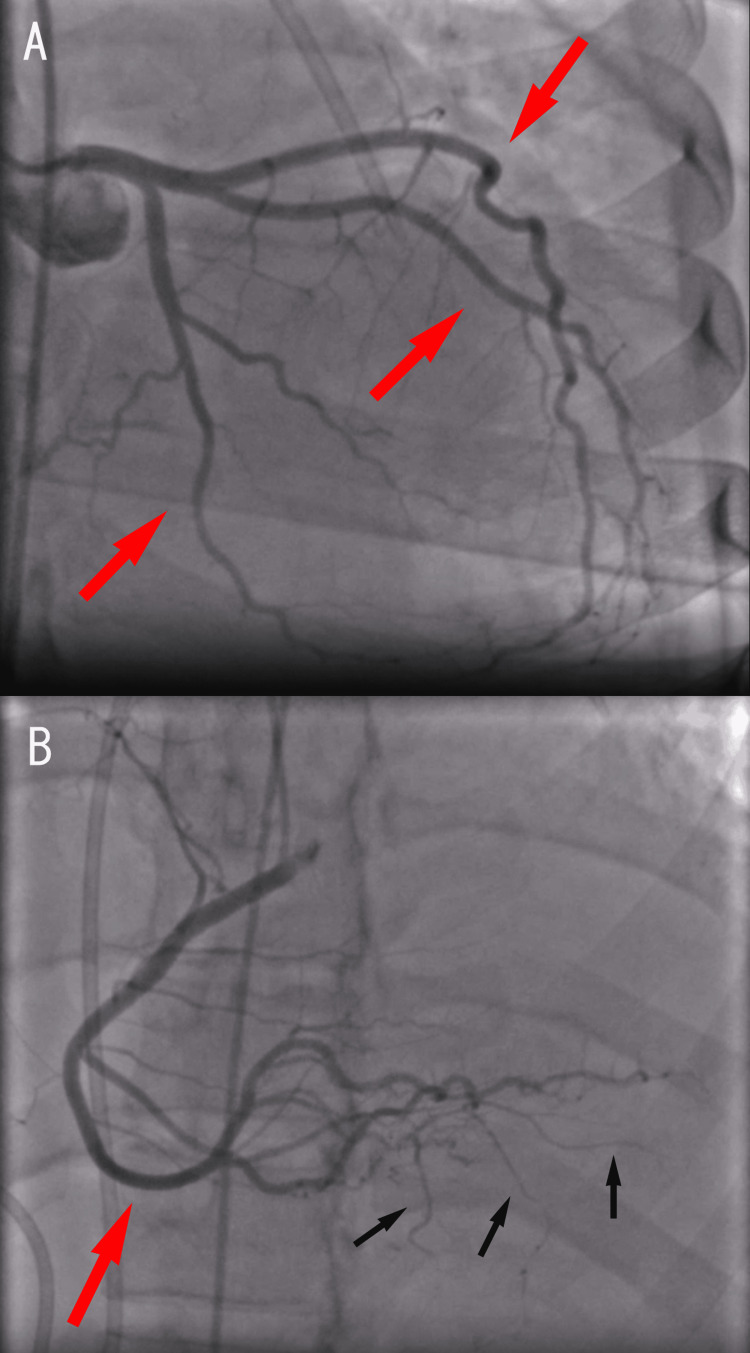
Left heart catheterization and coronary angiography (A) The left anterior oblique image showed normal coronary arteries without obstruction. (B) The right anterior oblique image showed normal right coronary arteries without obstruction (red arrow). It also showed increased vascularity at the distal aspect of the right coronary artery (black arrows).

STEMI alert was activated, and while preparing to transfer to the catheterization suite, the patient suffered cardiovascular collapse with pulseless electrical activity on EKG monitoring. Cardiopulmonary resuscitation was initiated, with a return of spontaneous circulation achieved in 20-30 seconds with no electrical defibrillation or epinephrine administration. The patient was transferred emergently to the catheterization suite, where a pericardial drain was placed with an immediate vigorous bloody output of 1,260 mL as well as normal coronary arteries on left heart catheterization. Increased vascularity was noted at the distal aspect of the right coronary artery, which would lead to the right ventricular (RV) apex (Figure 3).

**Figure 4:**
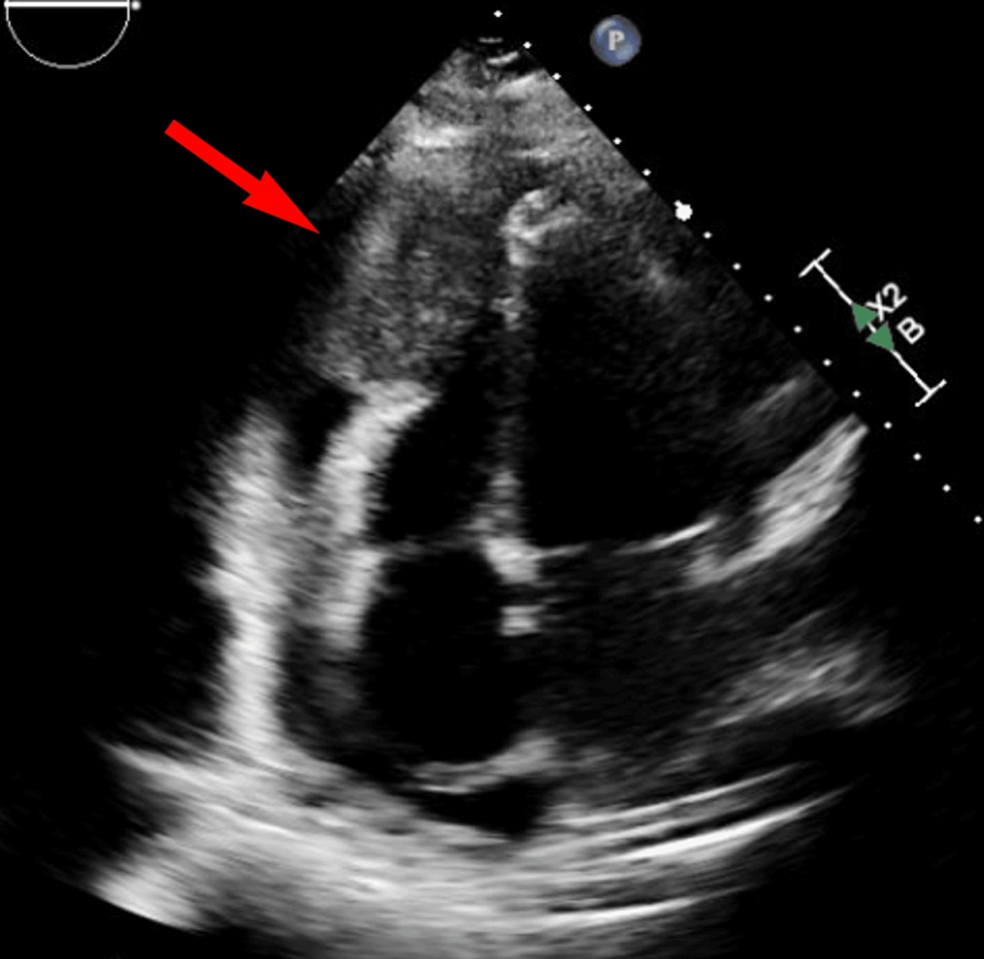
Transthoracic echocardiogram apical four-chamber view Transthoracic echocardiogram apical four-chamber view showed a heterogenous irregularly shaped mass adjacent to the right ventricle apex.

Initial fluid was sent for cytology and culture, which were initially negative for both malignancy and bacterial infection. The patient’s condition stabilized after drain placement, and the patient was transferred to the cardiac critical care unit for further management of pericardial tamponade. Over the next five days, the patient continued to have progressively decreasing drain output. Serial echocardiograms were obtained, demonstrating improved effusion, with persistent 4.5 cm x 4 cm heterogenous irregularly shaped mass visualized attached to the right ventricular apex (Figure 4).

**Figure 5:**
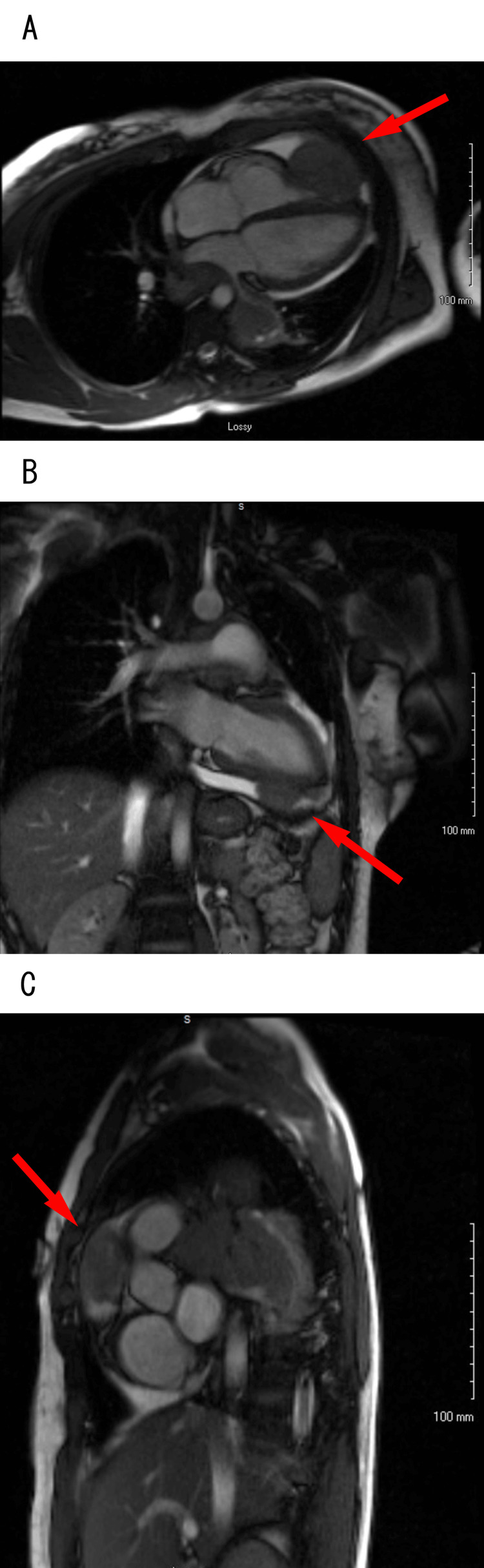
Cardiac magnetic resonance imaging Magnetic resonance imaging cine longitudinal relaxation time (T1) imaging of the (A) four-chamber view and (B) two-chamber view showed a 6 cm x 5 cm intrapericardial mass adjacent to the right ventricle apex without clear demarcation between the right ventricle free wall and mass. (C) Magnetic resonance imaging cine longitudinal relaxation time (T1) imaging of the short axis showed an intrapericardial mass anterior to the right ventricle outflow tract/pulmonary artery.

This would be the similar area that, prior to left heart catheterization, showed increased vascularity of the distal aspect of the right coronary artery. An immediate concern was for malignancy. Magnetic resonance imaging (MRI) head was negative for metastatic foci. Once drain output had diminished to zero, and echocardiographic stability of effusion was noted, the drain was removed, and cardiac MRI was obtained, which revealed multiple masses visualized in the pericardium, one mass anterior to the right ventricular outflow tract (RVOT)/pulmonary artery (PA) and a second mass adjacent to the RV apex, which was measured to be 6 cm x 5 cm (Figure 5).

**Figure 6:**
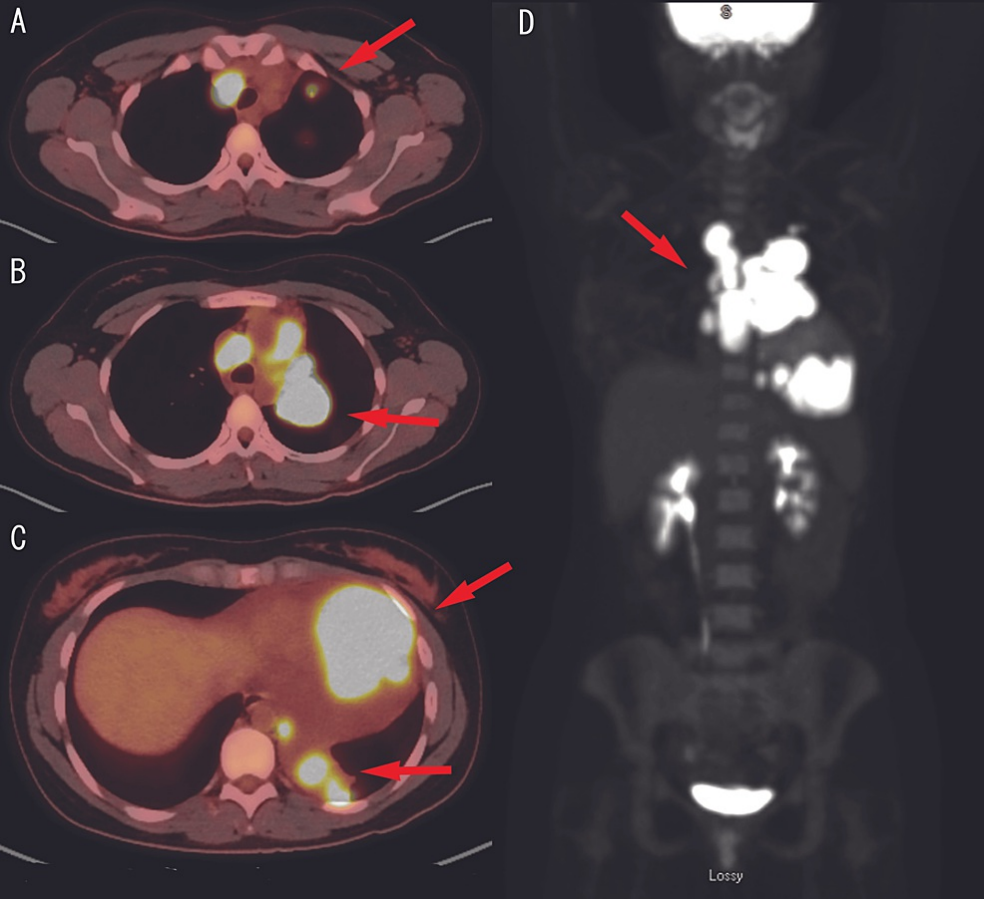
Positron emission tomography (tumor image) Positron emission tomography showed a (A) small hypermetabolic mass in the left upper lobe (standardized uptake values max of 8.7); (B) hypermetabolic central left hilar mass (standardized uptake values max of 15.6); (C) large hypermetabolic mass adjacent to the inferior apical border of the heart (standardized uptake values max of 15.8) and a hypermetabolic mass in the infrahilar left lower lobe (standardized uptake values max of 16.2); and (D) left intrahilar, left hilar, and left cardiophrenic masses with associated hypermetabolic right hilar, left hilar, subcarinal, costophrenic, aortopulmonary, paratracheal and mediastinal lymphadenopathy. No evidence of bone marrow lesions was noted.

Masses were hyperintense on longitudinal relaxation time (T1) sequence and had heterogenous late gadolinium enhancement. Given lung findings, masses were thought to be malignant; however, with no discernable margin between cardiac tissue and tumor, there was uncertainty on whether this was a primary cardiac malignancy or metastatic. The patient underwent positron emission tomography (Figure 6) scan, which revealed left intrahilar, hilar, and cardiophrenic masses with associated hypermetabolic right hilar, left hilar, subcarinal, costophrenic, aortopulmonary, paratracheal, and mediastinal lymphadenopathy. It also showed a hypermetabolic mass in the left upper and lower lobe of the lung. Computed tomography abdomen/pelvis showed no evidence of metastatic malignancy in the abdomen/pelvis. The patient subsequently underwent endobronchial ultrasound and a lymph node and lung mass biopsy, which initially resulted in a small round blue cell tumor. The biopsies showed an undifferentiated tumor composed of cells with round to ovoid nuclei, with high nucleus-to-cytoplasmic ratios and mild-to-moderate nuclear pleomorphism. The tumor cells are immunopositive for cluster of differentiation 99 (patchy, membranous), transducer-like enhancer of split 1 and desmin. Cytokeratin antigen extract 1/antigen extract 3 and cytokeratin 8/18 highlighted a few scattered columnar cells. After additional analysis, including next-generation sequence, the diagnosis was determined to be DSRCT with sarcoma fusion gene detected (EWSR-WT1). The first cycle of chemotherapy (doxorubicin 75 mg/m^2^ day one, vincristine 2 mg day one, cyclophosphamide 1,200 mg/m^2^ day one) was initiated, and the patient was transferred to an oncology center of excellence for further care.

## Discussion

Here, we reported a rare case of DSRCT presented with STEMI and cardiac tamponade with lung and pericardial masses. DSRCT typically presents with abdominal or retroperitoneal masses [3], and its tissue origin is still unknown [5]. DSRCT with lung mass was reported to be 1.56 % of the total cases in the Surveillance, Epidemiology, and End Results (SEER) database analysis [3].

This patient initially presented with inferolateral STEMI. However, a coronary angiogram showed no significant coronary artery stenosis. The patient was found to have a large pericardial effusion with cardiac tamponade secondary to intrapericardial tumor, which cardiac MRI noted multiple masses anterior to the RVOT/PA and adjacent to the RV apex. This is a rare condition where the etiology of STEMI is possible from alteration of cardiac oxygen consumption due to restricted coronary flow against high pericardial pressure [6] or tumor embolization. ST-segment elevation may present in patients with cardiac tamponade in only a small number of leads or contiguous leads [7,8]. This case highlights the significance of broad differential for ST changes on EKG besides myocardial infarction, especially in patients with young age and atypical presentation.

Given the clinical conditions, we assumed that her cardiac mass was also DSRCT, although the pathology of pericardial effusion was negative for malignant cells. Romanos-Sirakis et al. reported a case of cardiac DSRCT whose pericardial effusion was also negative for malignancy [9]. The presentation of the patient included dyspnea and abdominal pain that were similar to our case [9]. To the best of our knowledge, a few studies reported DSRCT pericardial masses [9-11], and their pathological results of pericardial effusions were not described.

We started the first cycle of chemotherapy that included doxorubicin 75 mg/m^2^ day one, vincristine 2 mg day one, and cyclophosphamide 1,200 mg/m^2^ day one, akin to Ewing sarcoma because the final pathological result was not revealed [12]. Cycle 2 was planned after the patient was transferred to an oncology center. The chemotherapy regimen for DSRCT usually consists of a combination of vincristine, doxorubicin, and cyclophosphamide alternating with ifosfamide and etoposide [5]. A randomized controlled trial regarding a chemotherapy regimen for DSRCT has not been performed given that DSRCT is a rare disease.

## Conclusions

DSRCT is a rare and aggressive disease that typically presents with abdominal or retroperitoneal masses with poor prognosis. Treatment includes chemotherapy, radiation, and cytoreductive surgery. We reported a case of DSRCT presented with STEMI and cardiac tamponade, found to have lung mass and pericardial masses. Left heart catheterization showed normal coronary arteries without obstruction. This case suggested that one of the differential diagnoses of lung and pericardium masses at a young age could be DSRCT. This case also highlighted the significance of broad differential for ST changes on EKG besides myocardial infarction, especially in patients with young age and atypical presentation.
